# The Interplay between Motivational, Affective Factors and Cognitive Factors in Learning: Editorial

**DOI:** 10.3390/jintelligence12070068

**Published:** 2024-07-19

**Authors:** Brenda R. J. Jansen

**Affiliations:** Department of Psychology, University of Amsterdam, 1018 WS Amsterdam, The Netherlands; b.r.j.jansen@uva.nl

Academic success is assumed to be both the start and outcome of a cycle in which affect, motivation, and effort strengthen each other (Vu et al. 2022). But how exactly do affective, motivational, and cognitive factors interact in learning? Ashcraft and Kirk’s (2001) experiments are exemplary demonstrations of the interplay between affective and cognitive factors in academic learning, in this case, mathematics anxiety and working memory. Through cleverly designed experiments, Ashcraft and Kirk showed that mathematics anxiety reduces working memory, which then cannot be used for solving a mathematics problem, thereby lowering mathematics performance. Through follow-up studies, including by other researchers and in younger age groups, it became increasingly clear that learners with different cognitive abilities (e.g., strong working memory) apply different problem-solving strategies, taxing working memory to varying extents, which made them more or less susceptible to mathematics anxiety. This affected mathematics tasks differently, dependent on the extent to which the tasks taxed working memory (for an overview, see, e.g., Beilock and Ramirez 2011). Eysenck et al. (2007) provide an extensive theoretical account and an overview of empirical evidence of how anxiety can impair performance, including in other academic domains.

This Special Issue presents studies that show an interplay between affective and cognitive factors in learning. Motivational factors were also considered, as motivation and academic emotions are tightly connected and are both related to academic performance (Pekrun 2006; Vu et al. 2022). This editorial presents a concise introduction to the studies and highlights some specific findings. 

The studies in this Special Issue show that the interplay between affective, motivational, and cognitive factors in learning can have different forms. Van der Ven et al. (2023) show an interaction between working memory and mathematics anxiety. That is, the negative relation between mathematics anxiety and mathematics performance strengthened with better working memory in one task. In other studies, the interplay is a mediating effect. That is, an affective or motivational factor is expected to result in effort (e.g., practice behaviour, investing time in school work), which should then be positively related to academic performance (Hilz et al. 2023; Liu et al. 2023). Other studies investigate factors that influence affect and motivation, such as the academic domain (Sasanguie et al. 2024), task demands (Kramer and Huizenga 2023; O’Connor et al. 2023; Van der Ven et al. 2023), school demands (Zhang and Jiang 2023), or academic buoyancy (Putwain et al. 2023). 

The empirical studies in this Special Issue were conducted in school settings, in children and adolescents. Some samples were impressively large, and some studies used data collected by school institutions as part of standard practice, for example, to monitor pupils’ performance (Hilz et al. 2023; Liu et al. 2023; O’Connor et al. 2023; Sasanguie et al. 2024; Van der Ven et al. 2023; Zhang and Jiang 2023). Studying the interplay between affective, motivational, and cognitive factors in the school setting itself, using actual monitoring data, promotes the generalisability of the results. In this way, experimental studies such as those by Ashcraft and Kirk (2001), which often took place in controlled settings with tasks specifically designed for the research question, are translated to daily school life. Such translations contribute to the relevance of the findings and enrich the field. 

Strikingly, half of the studies in this Special Issue concern the domain of learning mathematics and focus on mathematics anxiety. Of course, when a guest editor asks researchers in their network for contributions to a Special Issue, many contributors will be active in the same research area, in my case learning mathematics. Yet, contributions from other academic domains were explicitly asked for. The attention on mathematics anxiety highlights that mathematics is especially associated with (mostly negative) academic emotions and that motivation for mathematics is uncommon, at least in Western students. How can we explain mathematics’ bad name? Below is a list of reasons, which is by no means intended to be complete. First, mathematics requires the active use of working memory and other executive functions (Bull and Scerif 2001), making it a strenuous task. Second, mathematics problems require a multitude of skills, not only executive functions and basic number knowledge but also creativity in problem-solving. Relatedly, there is no fix-all solution for mathematics problems. Although various strategies can be correct, their efficiency depends on the mathematics problem at hand, and it takes experience to determine which strategy is both correct and efficient. Becoming better at mathematics thus involves time and effort. Third, mathematical elements build on each other, and understanding basic concepts is required to understand more complex concepts, making it difficult to catch up when missing or not understanding a concept introduced earlier. Perhaps because of these technical aspects, mathematics has become associated with certain beliefs, or so-called “math myths” (Barlow and Reddish 2006). These myths state that mathematics is not for all, that men are better in mathematics than women, or that “there is a magic key to doing math”, for example (Barlow and Reddish 2006, p. 149). Yet, whether such beliefs are universally endorsed is a topic for future research (Cvencek et al. 2011; see also this Special Issue: Liu et al. 2023; Zhang and Jiang 2023). 

This Special Issue shows that certain aspects of schools or academic tasks can worsen academic emotions and motivation. I discuss them from macro to micro levels. First, Zhang and Jiang (2023) mention that school demands may affect both satisfaction and frustration of the basic motivational needs of autonomy, relatedness, and competence. The authors note that the Chinese high school environment is quite demanding and competitive, which may explain why some students may focus on extrinsic goals and perceive low autonomy. Second, Sasanguie et al. (2024) highlight that anxiety is domain-specific. They show that mathematics performance is mainly related to mathematics anxiety, in comparison to reading anxiety and test anxiety. However, they do stress that academic anxieties are correlated and that one should be alert to anxiety in other academic domains when anxiety is seen in a particular academic domain. Third, task demands are related to academic emotions and motivation. Lengthy tasks lower motivation (Kramer and Huizenga 2023), and a high-stakes, complex mathematics task showed a stronger negative relation with performance than a low-stakes, simple mathematics task (Van der Ven et al. 2023). Also, mathematics anxiety had a stronger negative relation with mathematics performance in tasks that require application of the formal, symbolic number system, compared to non-symbolic tasks (O’Connor et al. 2023). 

The studies in this Special Issue show that the operationalisation of concepts is non-trivial. Although mathematics anxiety was operationalised similarly (based on the assumption that mathematics anxiety is a trait, using self-reports—even in young children), motivation, effort, and academic performance were operationalised differently. Motivation was assessed using self-reports but focused on the willingness to continue working on school work (Kramer and Huizenga 2023), value attached to education (Liu et al. 2023), self-concept (Hilz et al. 2023), or satisfaction and frustration with basic needs (Zhang and Jiang 2023). Effort was operationalised using a measure of practice behaviour frequency in an online practice programme (Hilz et al.) or self-reported time investment in learning (Liu et al.). Finally, performance was also operationalised differently. Kramer and Huizenga used a score on a test that might affect motivation, which was assessed right after the test. Other studies assessed performance in separate situations using a specific standardised test (Hilz et al.; O’Connor et al. 2023; Sasanguie et al. 2024; Van der Ven et al. 2023), self-rated performance (Liu et al.), or scores from schools’ monitoring or assessment systems (Liu et al., Sasanguie et al., Van der Ven et al., Zhang and Jiang). With such diverse operationalisations, different outcomes on the interplay between affective, motivational, and cognitive factors in learning are conceivable. 

The choice of statistical model is also non-trivial and may also result in different conclusions on the interplay. Liu et al. (2023) show that the dynamics of motivational and affective factors and performance can be described by classic cross-lagged panel models, as well as models with a random intercept or random curves, which control for stable, individual differences between learners. The statistical model determined whether a reciprocal relationship between motivation and performance was seen.

Individual differences in affect, motivation, achievement, and effort remain quite stable over time (Liu et al. 2023). This stability is surprising given that learners’ lives are full of events and daily “setbacks”, which may differ between individuals. Setbacks include disappointing results and dips in motivation (Putwain et al. 2023). A possible explanation for stability despite the individual differences in the dynamics of learners’ day-to-day lives is academic buoyancy: the extent to which an individual can bounce back after experiencing a setback. However, Putwain et al. show that results on the relation between academic buoyancy, and academic achievement are mixed. An important explanation for varying results may be that the timing of measuring setbacks, academic buoyancy, and academic performance is crucial. Setbacks, bouncing back, and academic performance recovery are unlikely to occur at the same time. Putwain et al. recommend time-intensive studies to capture these dynamics. 

Detecting significant relations in the interplay between affective, motivational, and cognitive factors in learning may be a starting point for interventions. Caution is however needed. Bailey et al. (2018) warn against intervening on early predictors of later skills, one reason being that the relation can be explained by underlying, unmeasured factors. For example, child–parent relations may play a role in both a child’s level of reported mathematics anxiety and mathematics performance (Casad et al. 2015), which might restrict the effects of a mathematics anxiety intervention on mathematics performance. Also, one must consider the selected operationalisation of the factors. For example, one performance intervention is an adaptive computerised practice programme that tailors problems to an individual’s ability, thereby levelling positive feedback for all pupils (Hilz et al. 2023; Jansen et al. 2013). However, this may not lower mathematics anxiety or boost mathematics self-concept because pupils also use information like class rank and exam scores to evaluate their performance, which may then feed mathematics anxiety and mathematics self-concept. 

An editorial is too short to do justice to all the individual studies. I invite the readers to dive into the studies that together offer an impressive array of research designs, age groups, and statistical techniques. All empirical studies took place in schools, complicating the implementation of a strict experimental design. The results convincingly show how the interplay between affective, motivational, and cognitive factors matters in everyday learning in schools.
